# COGNITIVE DECLINE ENHANCES VULNERABILITY TO ENVIRONMENTAL DETERMINANTS OF MOBILITY

**DOI:** 10.1093/geroni/igz038.1541

**Published:** 2019-11-08

**Authors:** Andrea Rosso, Philippa Clarke, Stephanie L Studenski, Caterina L Rosano

**Affiliations:** 1 University of Pittsburgh, Department of Epidemiology, Pittsburgh, Pennsylvania, United States; 2 university of michigan, Ann Arbor, Michigan, United States; 3 University of Pittsburgh, Pittsburgh, Pennsylvania, United States

## Abstract

Neighborhood and residential characteristics can potentially influence mobility of older adults but associations may differ based on cognitive function. We tested whether neighborhood and residential characteristics derived from audits of Google Streetview images were related to 4-year incident mobility disability and whether these associations differed by cognitive trajectories (maintainer vs decliner over 14 years). In 260 participants from the Health ABC (mean age=81.8 years, 57% female, 39% black), Cox proportional hazard models tested associations stratified by cognitive trajectory, adjusted for demographics. Mixed compared to residential land use was associated with greater risk of mobility disability among cognitive decliners (HR=1.27, 95% CI: 1.05-1.54) but not cognitive maintainers (HR=1.07, 95% CI: 0.90-1.28). Presence of slopes near the home and having a ramp at the home entrance were associated with lower mobility disability risk, again in decliners only. Lower cognitive function may increase vulnerability to poorer neighborhood and residential characteristics for mobility outcomes.

